# A Genetic Polymorphism in *pre-miR-27a* Confers Clinical Outcome of Non-Small Cell Lung Cancer in a Chinese Population

**DOI:** 10.1371/journal.pone.0079135

**Published:** 2013-11-06

**Authors:** Jiali Xu, Zhiqiang Yin, Hong Shen, Wen Gao, Yingying Qian, Dong Pei, Lingxiang Liu, Yongqian Shu

**Affiliations:** 1 Department of Oncology, The First Affiliated Hospital of Nanjing Medical University, Nanjing, China; 2 Department of Dermatology, The First Affiliated Hospital of Nanjing Medical University, Nanjing, China; MD Anderson Cancer Center, United States of America

## Abstract

**Background:**

Recent evidence indicates that microRNAs (miRNAs) can function as tumor suppressors and oncogenes. Single nucleotide polymorphisms (SNPs) at miRNA genes can influence the maturation of miRNAs or miRNA-mediated transcriptional regulation. Our objective was to investigate the association of SNPs in deregulated miRNAs with clinical outcome in patients with non-small cell lung cancer (NSCLC) in a Chinese population.

**Methods:**

Deregulated miRNAs in NSCLC and their SNPs were identified through public databases. A single SNP, rs895819 in *pre-miR-27a*, was found suitable for selection. TaqMan assays were performed for genotyping and to assess the effect on the overall survival (OS) and chemotherapy response in 576 NSCLC patients.

**Results:**

Log-rank test and Cox regression analysis indicated that the G allele of rs895819 was associated with shorter survival and increased risk of death in NSCLC [dominant model: 22.0 vs. 46.0 months, *P*<0.001; adjusted hazard ratio (HR) = 1.71, 95% confidential interval (CI): 1.12–2.26]. Further stepwise regression analysis suggested that this SNP was an independently unfavorable factor for the prognosis of NSCLC and the effect remained significant in subgroup analysis stratified by clinical parameters and treatment status. Moreover, multivariate logistic regression analysis showed that the subjects with AG/GG genotypes of rs895819 had significantly decreased response rate to platinum-based chemotherapy compared to those with the AA genotype.

**Conclusion:**

Our results suggest that the *pre-miR-27a* rs895819 polymorphism may influence NSCLC patients’ clinical outcome. Further large sample studies should be used to validate our findings.

## Introduction

Lung cancer is the leading cause of cancer-related deaths worldwide, due to its high incidence, malignant behavior and lack of effective in treatment strategy [1]. In China, there has been a significant increase in the incidence of lung cancer in both urban and rural areas over the last two decades, especially in non-small cell lung cancer (NSCLC) [2], [3]. Although platinum-based combination chemotherapy is the first-line treatment for patients with advanced NSCLC, the response rates vary among patients, ranging from 26% to 60% [4]. The five-year over survival (OS) rate is only 15% in the US and is even lower in China [1]. Established methods for predicting prognosis include the tumor, node, and metastasis (TNM) staging system, however, recent studies indicate that the discovery and application of specific prognostic biomarkers can improve the treatment and outcome of patients with NSCLC [5]. Despite a considerable amount of research [6], [7], [8], very few stable biomarkers have been identified for risk assessment or predication of clinical outcome and further investigations are necessary.

MicroRNAs (miRNAs) are a class of small endogenous noncoding RNAs that act as negative gene regulators by directly cleaving target mRNA or by inhibiting translation [9], [10]. The loss and gain of function of specific miRNAs or aberrant miRNA expression are thought to be key events in the tumorigenesis of many types of cancer [11], [12]. In NSCLC, miRNA expression profiles and specific miRNAs have been correlated with patients’ survival [13], [14]. In addition, there is increasing evidence that single nucleotide polymorphisms (SNPs) play a significant role in cancer susceptibility and outcome. The high degree of phylogenetic conservation in miRNA sequences determines that the functional genetic variations in miRNAs may affect various biological processes. Therefore, SNPs in miRNA genes could influence the primary transcripts (pri-miRNAs), precursor RNAs (pre-miRNAs), mature miRNAs or miRNA-mediated transcriptional regulation [15].

We utilized public databases to identify deregulated miRNAs in NSCLC and SNPs in these miRNAs sequences, including primary, precursor and mature miRNAs. Our searches identified a single SNP, rs895819 in *pre-miR-27a*, for further investigation. The rs895819 polymorphism is located in the terminal loop of *pre-miR-27a* and involves an A>G nucleotide transition. Hsa-miR-27a has been shown to function as an oncogene by targeting prohibitin [16] and is therefore a target for anticancer drugs [17], [18]. Metens-Talcott *et al*. [19] showed that transfection of antisense miR-27a in breast cancer cells led to an increase in expression of the putative Sp-repressor Zinc finger and BTB domain containing 10 (ZBTB10), with a concurrent decrease in the expression of Sp-dependent survival and angiogenic genes. These include vascular endothelial growth factor (VEGF) and survivin [19]. Over-expression of VEGF and surviving are associated with progression or poor survival of NSCLC [20], [21]. Sun *et al*. [22] reported rs895819 modifies gastric cancer susceptibility by modulating the expression levels of miR-27a and its target gene, *ZBTB10*. Shi *et al*. [23] suggested that this polymorphism could predict renal cell cancer risk in a Chinese population. Meta-analysis has shown that the *pre-mir-27a* polymorphism may play a role in breast cancer susceptibility and cancer development in Caucasian [24].

To date, there have been no reported studies on the relationship between *pre-mir-27a* polymorphisms and survival in cancer patients. Based on current knowledge of the biological functions of hsa-miR-27a and the role of polymorphisms in predicting cancer risk, we hypothesized that rs895819 might be associated with clinical outcome in NSCLC patients. Therefore, we evaluated the associations between rs895819 and NSCLC overall survival (OS), as well as response to platinum-based chemotherapy.

## Materials and Methods

### Ethics Statement

This study was approved by the institutional review board of Nanjing Medical University. All participants were voluntary and provided written informed consent prior to taking part in this research.

### Study Subjects

All subjects were recruited from the First Affiliated Hospital of Nanjing Medical University (Jiangsu, China) between January 2004 and September 2012. They were all newly diagnosed, histopathologically confirmed and without a prior history of cancer or previous chemo- or radiotherapy. In total, 612 patients with NSCLC were recruited, all of whom were unrelated ethnic Han Chinese population (CHB). A structured questionnaire on demographics and environmental exposure, including age, sex and smoking consumption, was conducted by trained interviewers through face-to-face interviews with the patients. In addition, 5 ml venous blood was collected from each patient for genomic DNA extraction. Subjects with a low frequency (<1 cigarette per day) and duration (<1 year) of smoking were defined as nonsmokers; all others were classified as smokers.

The response to platinum-based (cisplatin or carboplatin) chemotherapy in patients with advanced NSCLC was assessed following the the first two or three cycles and defined according to Response Evaluation Criteria in Solid Tumors (RECIST) criteria 1.1 [25]. The patients were divided into the following two groups based on their responses: those with complete response (CR) and partial response (PR) were classed as responders; those with stable disease (SD) and progressive disease (PD) were classed as non-responders. Follow-up was performed every three months from the time of enrollment until death or the last scheduled follow-up. The maximun follow-up time was 102.0 months (last follow-up in February 2013) and the medial follow-up time was 18.0 months. We selected the patients with complete follow-ups and adequate DNA sample. Patients who completed all the follow-ups and provided adequate DNA sample were selected. Of the original 612 patients, 576 were enrolled and genotyped in our study.

### Genotyping

Each blood sample was collected in an EDTA anticoagulant tube and stored at −80°C until DNA extraction. Genomic DNA was extracted following the standard protocols, with proteinase K digestion and phenol/chloroform extraction. Genotyping was performed by TaqMan allelic discrimination assays using an ABI 7900 system (Applied Biosystems, Foster City, CA, USA). The prime and probes are as follows: forward, GGCGGAACTTAGCCACTGT, reverse, CAGGGCTTAGCTGCTTGTG; FAM-ACTTGGTGTGGACC-MGB and HEX-ACTTGGCGTGGAC-MGB. The TaqMan assays were performed in a final reaction volume of 5 µl containing 0.25 µl primer, 0.125 µl probe, 2 µl PCR mixture reagent and 25 ng DNA. The PCR reaction consisted of an initial step at 95°C for 10 min followed by 55 cycles of denaturing at 95°C for 15 s and annealing at 60°C for 60 ss. SDS allelic discrimination software (ABI) was used to analyze the PCR genotyping results. Two blank (water) controls were included in each 384-well assay. At least 10% of samples were randomly selected for repeat analysis, yielding 100% concordance. A further 60 samples were selected randomly for direct sequencing to confirm the TaqMan results. Again, the results showed 100% concordance.

### Statistical Analysis

Hardy-Weinberg equilibrium was assessed by a goodness-of-fit χ^2^ test. OS was calculated as the time between the first treatment and death or the last follow-up date. Association between the genotype and survival rate was estimated by the Kaplan–Meier method and log-rank test. The median survival time (MST) was also calculated. Cox proportional hazards models were performed to estimate the hazard ratios (HRs) and their 95% confidential intervals (CIs) for OS. Cox stepwise regression model was also conducted to determine predictive factors to NSCLC prognosis. The *P* value for the heterogeneity test was based on the χ^2^-based Q test. Odds ratios (ORs) and their 95% CIs were calculated as a measure of difference in the response rate using logistic regression analysis (responders vs. non-responders). All statistical analyses were performed using SPSS 18.0 software (SPSS Inc., Chicago, Illinois, USA), and *P*<0.05 based on a two-side test was considered statistically significant.

## Results

### Characteristics of the Study Population

**Figure 1:**
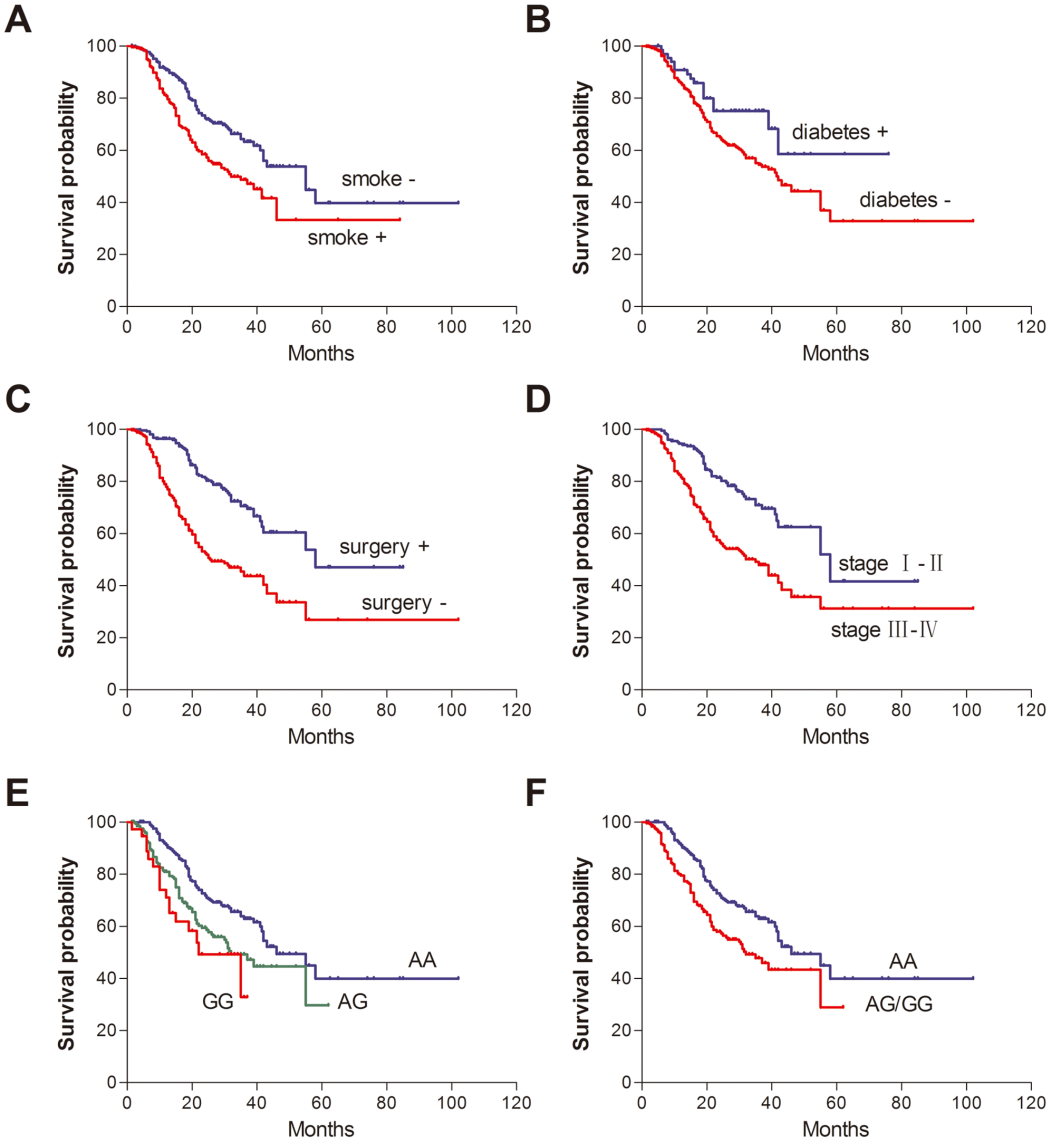
Kaplan-Meier plots of overall survival by clinical parameters, treatment status and *pre-miR-27a* rs895819 genotypes. Log-rank *P* values were (A) <0.001, (B) 0.034, (C) <0.001, (D) <0.001, (E) <0.001 and (F) <0.001, respectively.

The demographic and clinical characteristics of the 576 patients enrolled in this study, and their association with OS are given in Table S1. The median age at diagnosis was 60 years (range, 29–86 years), and included 380 males (66.0%) and 267 smokers (46.4%). Among these patients, 381 (66.2%) were adenocarcinomas, 166 (28.8%) were squamous cell carcinomas, and the others (29 patients, 5.0%) were large cell, undifferentiated or mixed-cell carcinomas. During the follow-up period, 206 patients died from NSCLC. Smoking status, clinical stage and surgical operation were significantly associated with survival time (all log-rank *P*<0.001, Figure 1); however, chemotherapy and targeted therapy were not. Of note, patients with diabetes had a 42% significantly decreased risk of death, compared with those without diabetes (54.9 *vs*. 42.0 months; HR = 0.58, 95% CI: 0.35–0.97).

### SNP Selection

The IGDB.NSCLC database (http://igdb.nsclc.ibms.sinica.edu.tw) [26] was used to identify deregulated miRNAs in NSCLC. A total of 28 up-regulated miRNAs in amplified regions and 28 down-regulated miRNAs in deleted regions were found. Patrocles database (www.patrocles.org) [27] identified 5 miRNAs with 5 putative SNPs within the pre-miRNAs or mature miRNAs sequences of these 56 miRNAs (Table 1). These included rs2043556 within the *pre-miR-605* sequence and rs895819 within the *pre-miR-27a* sequence, which both had a minor allelic frequency (MAF) >5% in CHB. The rs2043556 was excluded because the high GC content can lead to failure in the TaqMan probe assay. Therefore, only one SNP, rs895819 A>G, was finally selected.

**Table 1:** Putative SNPs in miRNA sequences.

Expression status in NSCLC	miRNA	location	polymorphism	Frequency in CHB
up	mir-299	mature	rs41286566	NA^a^
up	mir-605	premature	rs2043556	0.321
down	mir-27a	premature	rs11671784	NA
down	mir-27a	premature	rs895819	0.314
down	mir-34a	mature	rs35301225	NA

a NA, not available.

### Association between the *pre-miR-27a* Polymorphism and Survival in NSCLC

The genotype frequencies of the rs895819 were in Hardy-Weinberg equilibrium (*P* = 0.538). The Log-rank test showed a significant association between rs895819 and survival in patients with NSCLC in the codominant and dominant models (both *P*<0.001; Figure 1). Patients carrying at least one variant allele had a shorter OS compared to those with the AA genotype (22.0 *vs.* 46.0 months, *P*<0.001). Univariate Cox regression analysis showed that this SNP was a significant prognostic marker of NSCLC (dominant model: HR = 1.74, 95% CI: 1.32–2.28). The association remain significant after adjustment for age, sex, smoking status, diabetes mellitus, histology, clinical stage, surgical operation and treatment status (dominant model: HR = 1.71, 95% CI: 1.12–2.26; Table 2).

**Table 2:** Genotypes of rs895819 polymorphism and NSCLC patients’ survival.

Genotype	PatientsN (%)	DeathsN (%)	MST(months)	Log-rank*P*	Crude HR(95% CI)	*P*	Adjusted HR(95% CI)^a^	*P*
AA	332(57.6)	104(31.3)	46.0	<0.001	1.00		1.00	
AG	207(35.9)	85(41.1)	32.0		1.66(1.25–2.22)	0.001	1.72(1.28–2.31)	<0.001
GG	37(6.4)	17(45.9)	22.0		2.24(1.34–3.75)	0.002	1.66(0.98–2.80)	0.059
Allelic trend					1.56(1.27–1.93)	<0.001	1.44(1.17–1.77)	0.001
AA	332(57.6)	104(31.3)	46.0	<0.001	1.00		1.00	
AG/GG	224(42.4)	102(41.8)	32.0		1.74(1.32–2.28)	<0.001	1.71(1.12–2.26)	<0.001

a Adjusted for age, sex, smoking status, diabetes mellitus, histology, clinical stage, surgical operation and treatment status.

In order to identify independent prognostic factors for NSCLC survival, we performed multivariate stepwise Cox regression analysis using selected demographic characteristics, clinical features and the *pre-miR-27a* polymorphism. The final predictive model included the SNP rs895819 (*P*<0.001), along with smoking status (*P* = 0.018), diabetes mellitus (*P* = 0.042) and surgery (*P*<0.001) (Table 3).

**Table 3:** Results of multivariate Cox regression analysis on NSCLC patients’ survival.

Variables	β	SE	HR	95% CI	*P*
Stepwise regressionanalysis					
Sex (femalevs. male )	−0.312	0.213	0.73	0.48–1.11	0.141
Smoking status (evervs. never)	0.456	0.193	1.58	1.08–2.30	0.018
Diabetes mellitus (yesvs. no)	−0.533	0.263	0.59	0.35–0.98	0.042
Clinical stage (III–IVvs. I–II)	0.196	0.250	1.22	0.76–1.98	0.433
Surgical operation(yes vs. no)	−0.969	0.236	0.38	0.24–0.60	<0.001
Rs895819(AG/GG vs. AA)	0.555	0.143	1.74	1.32–2.31	<0.001

### Stratification and Interaction Analysis

The association between *pre-miR-27a* polymorphism and NSCLC survival was further evaluated by stratified analysis of age, sex, smoking status, diabetes mellitus, histology, clinical stage, surgical operation, chemotherapy and targeted therapy. As shown in Table 4, an increased risk of death was observed in the following subjects: older subjects (adjusted HR = 2.71, 95% CI: 1.84–4.00); males (adjusted HR = 2.05, 95% CI: 1.46–2.87); smokers (adjusted HR = 2.08, 95% CI: 1.40–3.08); those without diabetes (adjusted HR = 1.53, 95% CI: 1.15–2.05); those with adenocarcinomas (adjusted HR = 1.73, 95% CI: 1.19–2.49); those who did not undergo surgery (adjusted HR = 1.67, 95% CI: 1.18–2.36); received chemotherapy (adjusted HR = 1.66, 95% CI: 1.23–2.25); and have not received targeted-therapy (adjusted HR = 1.71, 95% CI: 1.26–2.32). Heterogeneity test between each pair of strata showed that heterogeneity was significant for smoking status (*P* = 0.019). However, gene-smoking interaction analysis revealed no significant interaction between the rs895819 polymorphism and smoking status (data not shown).

**Table 4:** Stratified analysis for rs895819 genotypes and NSCLC patients’ survival.

Variables	Deaths/patients		Rs895819(deaths/patients)								
			AA		AG+GG		Crude HR(95% CI)	*P*	Adjusted HR(95% CI)^a^	*P*	*P* b
			N	%	N	%					
Total	206/576		104/332	50.5/57.6	102/224	49.5/42.4	1.74(1.32–2.28)	<0.001	1.71(1.12–2.26)	<0.001	
Age											0.691
≤60	92/273		53/155	57.6/56.8	39/118	42.4/43.2	1.14(0.75–1.72)	0.547	1.02(0.67–1.56)	0.920	
>60	114/303		51/177	44.7/58.4	63/126	55.3/41.6	2.48(1.71–3.60)	<0.001	2.71(1.84–4.00)	<0.001	
Sex											0.480
Male	147/380		70/223	47.6/58.7	77/157	52.4/41.3	2.11(1.52–2.92)	<0.001	2.05(1.46–2.87)	<0.001	
Female	59/196		34/109	57.6/55.6	25/87	42.4/44.4	1.20(0.71–2.02)	0.497	1.19(0.67–2.09)	0.558	
Smoking status											0.019
Never	94/309		56/192	59.6/62.1	38/117	40.4/37.9	1.28(0.85–1.94)	0.235	1.21(0.78–1.87)	0.395	
Ever	112/267		48/140	42.9/52.4	64/127	57.1/47.6	2.17(1.48–3.17)	<0.001	2.08(1.40–3.08)	<0.001	
Diabetes mellitus											0.320
None	190/508		99/289	52.1/56.9	91/219	47.9/43.1	1.48(1.11–1.97)	0.007	1.53(1.15–2.05)	0.004	
Yes	16/68		5/43	31.3/63.2	11/25	68.8/36.8	12.9(3.51–47.5)	<0.001	–	–	
Histology											0.699
Adenocarcinoma	127/381		65/218	51.2/57.2	62/163	48.8/42.8	1.66(1.17–2.36)	0.005	1.73(1.19–2.49)	0.004	
Squamous Cell	71/166		38/99	53.5/59.6	33/67	46.540.4	1.85(1.15–2.97)	0.011	1.34(0.81–2.20)	0.254	
Others^c^	8/29		1/15	12.5/51.7	7/14	87.5/48.3	7.69(0.94–63.0)	0.058	–	–	
Clinical stage											0.599
I	21/104		11/66	52.4/63.5	10/38	47.6/36.5	1.89(0.80–4.45)	0.147	1.32(0.50–3.48)	0.573	
II	32/97		17/65	53.1/56.7	15/42	46.9/43.3	1.28(0.63–2.60)	0.492	1.41(0.63–3.15)	0.400	
III	38/102		17/56	44.7/54.9	21/46	55.3/45.1	2.23(1.17–4.25)	0.014	1.58(0.71–3.51)	0.261	
IV	115/273		59/155	51.3/56.8	56/118	48.7/43.2	1.75(1.20–2.54)	0.004	1.42(0.97–2.10)	0.074	
Surgical operation											0.315
None		140/326	70/182	50.0/55.8	70/144	50.0/44.2	1.77(1.26–2.49)	0.001	1.67(1.18–2.36)	0.004	
Yes		66/250	34/150	51.5/60.0	32/100	48.5/40.0	1.69(1.04–2.74)	0.033	1.54(0.94–2.54)	0.090	
Chemotherapy											0.288
None		26/55	11/28	42.3/50.9	15/27	57.7/49.1	1.73(0.79–3.79)	0.172	2.42(0.80–7.37)	0.119	
Yes		180/521	93/304	51.7/58.3	87/217	48.3/41.7	1.72(1.28–2.31)	<0.001	1.66(1.23–2.25)	0.001	
Targeted therapy											0.407
None		174/480	85/273	48.9/56.9	89/207	51.5/43.1	1.77(1.32–2.39)	<0.001	1.71(1.26–2.32)	0.001	
Yes		32/96	19/59	59.4/61.5	13/37	40.6/38.5	1.55(0.76–3.16)	0.230	1.47(0.63–3.43)	0.375	

a Adjusted for age, sex, smoking status, diabetes mellitus, histology, clinical stage, surgical operation and treatment status.

b *P* value for heterogeneity.

c Other carcinomas include large cell, undifferentiated and mixed-cell carcinomas.

### Association between the *pre-miR-27a* Polymorphism and Response to Platinum-based Chemotherapy

The association between rs895819 and treatment response was further evaluated. Only the 296 patients with inoperable advanced stage NSCLC (IIIb–IV), who had received platinum-based chemotherapy as first-line therapy, and had a complete evaluation of chemotherapy response, were included to avoid potential confounding effects from surgery, clinical stage and treatment type. Detailed treatment information is provided in Table S2. The multivariate logistic regression model showed that patients with a G allele of rs895819 had significantly decreased response rate to platinum-based chemotherapy compared to those with the AA genotype (AG/GG vs. AA: adjusted OR = 0.54, 95% CI = 0.32–0.91) and consequently, an increased risk of death (adjusted HR = 1.59, 95% CI: 1.09–2.31). We detected the similar results using an additive model (adjusted OR = 0.54, 95% CI = 0.35–0.83; adjusted HR = 1.59, 95% CI: 0.97–1.63; Table 5).

**Table 5:** Genotypes of rs895819 polymorphism and the association with platinum-based chemotherapy response and OS in patients with advanced NSCLC.

Genotype	Response							OS			
	PatientsN (%)		RespondersN (%)	Crude OR(95% CI)	*P*	Adjusted OR(95% CI) a	*P*	DeathsN (%)	MST(Months)	Adjusted HR(95% CI) a	*P*
Rs895819											
AA	168(56.8)		68(40.5)	1.00		1.00		60(35.7)	43.0	1.00	
AG	103(34.8)		30(29.1)	0.60(0.39–1.02)	0.060	0.65(0.37–1.12)	0.120	50(48.5)	21.0	1.71(1.15–2.53)	0.007
GG	25(8.4)		3(12.0)	0.20(0.06–0.70)	0.011	0.19(0.05–0.68)	0.010	12(48.0)	15.0	1.20(0.63–2.29)	0.584
Allelic trend				0.53(0.35–0.80)	0.003	0.54(0.35–0.83)	0.005			1.26(0.97–1.63)	0.090
AA	168(56.8)		68(40.5)	1.00		1.00		60(35.7)	43.0	1.00	
AG/GG	128(43.2)		33(25.8)	0.51(0.31–0.84)	0.009	0.54(0.32–0.91)	0.020	62(48.4)	21.0	1.59(1.09–2.31)	0.016

a Adjusted for age, sex, smoking status, diabetes mellitus, histology, clinical stage, surgical operation and treatment status.

## Discussion

In the present study, we found that the variant allele G of rs895819 within *pre-miR-27a* was associated with a significantly increased risk of deaths for NSCLC. The effect was even stronger in older subjects, males, those with adenocarcinomas, without diabetes, and in those who did not undergo surgical operation and have received chemotherapy. Furthermore, a significantly decreased response rate to platinum-based chemotherapy was observed in advanced NSCLC patients with AG/GG genotypes. This is the first reported study on the association between the *pre-miR-27a* rs895819 polymorphism and the clinical outcome in patients with NSCLC.

Although miRNAs represent only a small part of the genome, they regulate almost one-third of human genes [28]. An increasing number of studies have indicated that miRNAs are involved in critical biological processes, including development, differentiation, apoptosis and proliferation, and may therefore play important roles in carcinogenesis as tumor oncogenes or suppressors [29], [30]. Genetic polymorphisms in miRNAs have been reported to be associated with many tumors, including gastric cancer [31], breast cancer [32] and colorectal cancer [33]. Hu *et al*. [14] found that a variant homozygote of rs11614913 in *pre-miR-196a2* was associated with poor survival in patients with NSCLC. Hong *et al*. [34] suggested that the *pre-miR-149* rs2292832 T>C polymorphisms were significantly associated with disease-free survival and OS in NSCLC. Moreover, genetic variations within the miRNA-machinery genes or miRNA-binding sites in 3′-untranslated region of target genes have also been reported to be associated with cancer susceptibility or prognosis [35], [36], [37].

It has been suggested that a genetic variation causing structural change in a critical region of miRNA may affect the process and the maturation of miRNA [38], [39]. The *pre-miR-196a2* rs11614913 C allele was associated with significantly increased expression of mature miR-196a and could therefore affect the binding of miR-196a2 to its target mRNA *LSP1* [14], [40]. The SNP rs11671784 has been reported to impair the maturation of miR-27a, resulting in reduced expression of mature miR-27a and an increased level of its target *HOXA10* [41]. The SNP rs895819 has also been indentified in pre-miRNA regions of hsa-miR-27a, with a MAF >5% in CHB. Preliminary functional assays by Sun *et al*. [22] revealed that the rs895819 G allele conferred a higher level of miR-27a, which accompanied significantly reduced *ZBTB10* mRNA. We used the RNAfold program to predict the most stable secondary structures of *pre-miR-27a* with two different alleles. However, neither a conformational effect nor a free energy effect was predicted (data not shown). In contrast, on the basis of the model of Zeng and Cullen, mutations in the stem of pre-miRNAs had a marked effect on miRNAs processing [39]. Although the SNP within miR-27a was located in the terminal loop, the mutant within terminal loop may slightly impair pri-miRNA processing and DGCR8 binding based on the ssRNA-dsRNA Junction Anchoring Model [42]. Such a mechanism has been demonstrated for hnRNP A1 [43].

We found that the G allele of rs895819 within *pre-miR-27a* was associated with a significantly decreased response rate to platinum-based chemotherapy and a significantly increased risk of deaths for NSCLC. Hsa-miR-27a has been found to be overexpressed in several types of tumors, including breast cancer [44] and ovarian cancer [45]. It has been shown to function as an oncogene by targeting prohibitin [16] and is a target for anticancer drugs [17], [18]. Down-regulated miR-27a might can also reverse multidrug resistance of esophageal squamous cell carcinoma [46]. Although the expression of miR-27a has been reported to be significantly reduced in both tissue and serum samples of NSCLC patients [26], [47], a relatively higher level of miR-27a expression was observed with the rs895819 G allele compared to the A allele [22]. These evidence may partly support our findings. Further in-depth functional studies are required to elucidate the mechanism of miR-27a and this variant in NSCLC.

We also observed an increase in OS in patients with both NSCLC and diabetes mellitus compared to those without diabetes (54.9 vs. 42.0 months; *P* = 0.034). Multivariate stepwise Cox regression analysis showed that this was an independent prognostic factor for NSCLC, which is consistent with several cohort studies in Caucasians [48], [49]. Oral antidiabetic drugs, especially metformin, are commonly administered as front-line treatment for patients with diabetes. Both basic and animal experiments have indicated that a metformin-mediated reduction in insulin resistance may be related to a reduced risk of tumor development [50], [51], [52]. Metformin administered alone or in combination with chemotherapy has also been reported to block tumor growth *in vitro* and *in vivo* experiments [53], [54], [55]. The findings of Tan *et al*. [56] demonstrated that metformin may improve chemotherapy outcomes and survival for NSCLC patients with diabetes. These studies support our results.

When interpreting our results, several limitations should be concerned. Firstly, all of the subjects were recruited from a single institution, which can introduce selection bias. Nonetheless, the genotype distribution was in Hardy-Weinberg equilibrium. Secondly, our sample size was relatively small. However, we achieved 89% study power (two-sided test, α = 0.05) to detect an HR of 1.71 for the rs895819 G genotype in dominant model. Thirdly, due to lack of lung cancer tissue corresponding to the blood specimen analyzed, we were unable to detect the expression of miR-27a and its targets, and explore the association between them and SNP rs895819. Despite these limitations, this is the first study to investigate the association between the *pre-miR-27a* rs895819 polymorphism and clinical outcome of NSCLC, and provided valuable information which might serve to guide future studies and clinical practice.

## Conclusions

In conclusion, our results provided the first insight into the contribution of *pre-miR-27a* rs895819 polymorphism to the clinical outcome of NSCLC. The findings further highlight that polymorphisms in miRNA sequences may play an important role in lung cancer. Although the association appeared to be statistically significant in the present study, the findings should be further validated by large, well-designed studies.

## Supporting Information

Table S1 Patient characteristic and clinical features.
(DOC)

Table S2 Treatment characteristics of the 296 patients.
(DOC)
