# Supplementary material for: A Genetic Polymorphism in pre-miR-27a Confers Clinical Outcome of Non-Small Cell Lung Cancer in a Chinese Population
Source: PLoS One. 2013 Nov 6;8(11):e79135. doi: 10.1371/journal.pone.0079135 (PMC3819265; doi:10.1371/journal.pone.0079135)
Supplement: Table S1 — Patient characteristic and clinical features. (DOC) [file pone.0079135.s001.doc]

**Table S1.** Patient characteristic and clinical features.

| Variables | Patients | Deaths | MST (mo) | Log-rank *P* | HR (95% CI) |
| --- | --- | --- | --- | --- | --- |
|  | n=576 (%) | n=206 |  |  |  |
| Age |  |  |  | 0.350 |  |
| ≤60 | 273 (47.4) | 92 | 53.2^a^ |  | 1.00 |
| >60 | 303 (52.6) | 114 | 42.0 |  | 1.14 (0.87-1.50) |
| Sex |  |  |  | 0.002 |  |
| Male | 380 (66.0) | 147 | 39.0 |  | 1.00 |
| Female | 196 (34.0) | 59 | 55.0 |  | 0.63 (0.46-0.85) |
| Smoking status |  |  |  | <0.001 |  |
| Never | 309 (53.6) | 94 | 55.0 |  | 1.00 |
| Ever | 267 (46.4) | 112 | 32.0 |  | 1.76 (1.34-2.32) |
| [Diabetes mellitus](http://dict.cn/diabetes mellitus) |  |  |  | 0.034 |  |
| None | 508 (88.2) | 190 | 42.0 |  | 1.00 |
| Yes | 68 (11.8) | 16 | 54.9^a^ |  | 0.58 (0.35-0.97) |
| Histology |  |  |  | 0.071 |  |
| Adenocarcinoma | 381 (66.2) | 127 | 55.0 |  | 1.00 |
| Squamous Cell | 166 (28.8) | 71 | 36.0 |  | 1.40 (1.05-1.87) |
| Others^b^ | 29 (5.0) | 8 | 40.8^a^ |  | 1.07 (0.52-2.18) |
| Clinical stage |  |  |  | <0.001 |  |
| Ⅰ | 104 (18.1) | 21 | 65.4^a^ |  | 1.00 |
| Ⅱ | 97 (16.8) | 32 | 55.0 |  | 1.96 (1.13-3.41) |
| Ⅲ | 102 (17.7) | 38 | 39.0 |  | 2.51 (1.47-4.28) |
| Ⅳ | 273 (47.4) | 115 | 31.0 |  | 3.69 (2.31-5.90) |
| Surgical operation |  |  |  | <0.001 |  |
| None | 326 (56.6) | 140 | 26.0 |  | 1.00 |
| Yes | 250 (43.4) | 66 | 58.0^a^ |  | 0.37 (0.27-0.50) |
| Chemotherapy |  |  |  | 0.079 |  |
| None | 65 (9.5) | 26 | 31.5 |  | 1.00 |
| Yes | 521 (90.5) | 180 | 43.0 |  | 1.44 (0.95-2.18) |
| Targeted therapy |  |  |  | 0.058 |  |
| None | 480 (83.3) | 174 | 46.0 |  | 1.00 |
| Yes | 96 (16.7) | 32 | 42.0 |  | 0.70 (0.48-1.02) |

^a^ Mean survival time was provided when MST could not be calculated.

^b^ Other carcinomas include large cell, undifferentiated and mixed-cell carcinomas.
