# Supplementary material for: A Genetic Polymorphism in pre-miR-27a Confers Clinical Outcome of Non-Small Cell Lung Cancer in a Chinese Population
Source: PLoS One. 2013 Nov 6;8(11):e79135. doi: 10.1371/journal.pone.0079135 (PMC3819265; doi:10.1371/journal.pone.0079135)
Supplement: Table S2 — Treatment characteristics of the 296 patients. (DOC) [file pone.0079135.s002.doc]

Table S2. Treatment characteristics of the 296 patients.

| Chemotherapy regimens | Patients (N%) |
| --- | --- |
| DDP/CBP + TAX/TXT/DOC | 159 (53.7) |
| DDP/CBP + GEM | 73 (24.7) |
| DDP/CBP + Pemetrexed | 53 (17.9) |
| DDP/CBP + NVB | 11 (3.7) |

DDP, cisplatin; CBP, carboplatin; TAX, taxol/paclitaxel; TXT, tanetere; DOC, docetaxel; GEM, gemcitabine; NVB, vinorelbine. The concrete dosage were as follows: DDP 75 mg/m^2^ on Day 1; CBP AUC 5-6 g on Day 1; taxol 175 mg/m^2^ on Day 1 (kept for 3h); taxotere 75 mg/m2 on Day 1 (kept for 1h); docetaxel 60 mg/m2 on Day1 (kept for 1h); gemcitabine 1250 mg/m^2^ on Days 1 and 8; vinorelbine 25 mg/m^2^ on Days 1 and 8; pemetrexed 800 mg/m^2^ on Day 1. All the chemotherapeutic agents were administered intravenously.
